# Dealing with locally-driven degradation: A quick start option under REDD+

**DOI:** 10.1186/1750-0680-6-16

**Published:** 2011-12-28

**Authors:** Margaret M Skutsch, Arturo Balderas Torres, Tuyeni H Mwampamba, Adrian Ghilardi, Martin Herold

**Affiliations:** 1 Centro de Investigaciones en Geografía Ambiental, Universidad Nacional Autonoma de Mexico, Campus Morelia, Antigua Careterra a Patzcuaro 8710, CP 58190, Morelia, Mexico; 2 Twente Centre for Studies in Technology and Sustainable Development, University of Twente, PO Box 217, 7500AE Enschede, the Netherlands; 3 Ingenieria Ambiental, PTI Instituto Tecnológico y de Estudios Superiores de Occidente (ITESO), Tlaquepaque CP 45090 México; 4 Wageningen University. Center for Geoinformation, Droevendaalsesteeg 3, 6708 PB Wageningen, the Netherlands

**Keywords:** Low-level degradation, below canopy, community management, monitoring, RELs, RLs, crediting, nesting.

## Abstract

The paper reviews a number of challenges associated with reducing degradation and its related emissions through national approaches to REDD+ under UNFCCC policy. It proposes that in many countries, it may in the short run be easier to deal with the kinds of degradation that result from locally driven community over-exploitation of forest for livelihoods, than from selective logging or fire control. Such degradation is low-level, but chronic, and is experienced over very large forest areas. Community forest management programmes tend to result not only in reduced degradation, but also in forest enhancement; moreover they are often popular, and do not require major political shifts. In principle these approaches therefore offer a quick start option for REDD+. Developing reference emissions levels for low-level locally driven degradation is difficult however given that stock losses and gains are too small to be identified and measured using remote sensing, and that in most countries there is little or no forest inventory data available. We therefore propose that forest management initiatives at the local level, such as those promoted by community forest management programmes, should monitor, and be credited for, only the net increase in carbon stock over the implementation period, as assessed by ground level surveys at the start and end of the period. This would also resolve the problem of nesting (ensuring that all credits are accounted for against the national reference emission level), since communities and others at the local level would be rewarded only for increased sequestration, while the national reference emission level would deal only with reductions in emissions from deforestation and degradation.

## 1. Introduction

Degradation - the (anthropogenic) loss of biomass in 'forests which remain forests' [1] - is one of the five components included in international policy on Reduced Emissions from Deforestation and Degradation (REDD+), the others being deforestation, forest enhancement, sustainable management of forests (SFM) and conservation [2]. In many ways, degradation is the least understood component of REDD+. In the literature on REDD+, 'degradation' is often implicitly used to refer to the effects of selective logging (legal or illegal) in humid tropical forests [3-6]. This is only one of the processes by which degradation occurs however. Low level chronic degradation occurs in a variety of forest types, and is quite possibly a much larger contributor to global carbon emissions than degradation by selective logging, as it occurs over much wider areas. Low-level degradation results from continuous over-exploitation of forests by communities for their livelihood needs, particularly for grazing, but also for shifting cultivation and in some places for fuel. It is more prevalent and widespread in dry forests than in rainforest because of the higher population densities of these areas [7]. A third major cause of degradation, besides commercial selective logging and local-driven low level chronic degradation, is escaped manmade fire. This may occur in a variety of forest types, and is particularly difficult to deal with in REDD+ because in some ecosystems, fire is a natural element and essential for their long run maintenance [8].

Although these major types of degradation can be easily recognized, it has proven very difficult to define degradation in terms which can be used in the international agreements, and more importantly, which allow measurement and carbon accounting.

This paper provides a short review of the technical challenges involved in defining degradation specifically for the purposes of REDD+, and suggests an approach that would enable countries to start measuring the carbon impacts of programmes dealing with emissions from degradation, in particular by addressing locally-driven degradation associated with subsistence use of forest by local communities. Further, we argue that forest enhancement as a component in REDD+ is for all practical purposes 'negative' (or 'reversed') degradation, and a direct result of improved management. It can and should be measured using the same metrics as those used for degradation.

We go on to suggest that in the short term it may be easier to combat the kinds of degradation that are related to local community use of forests than those associated with selective logging or with fire. This can be done through programmes of community management, which offer a route for countries to get to grips with at least those degradation emissions which are community-induced, rather quickly. We discuss the data requirements for this, particularly as regards developing reference emissions levels/reference levels (RELs/RLs)^a ^both at the local (management unit) and the national^b ^level.

We conclude with some innovative suggestions about how crediting for this approach could be organized. Having shown that, for a variety of reasons, it will be very difficult in the short term to construct a REL either at local or at national level for degradation, we propose a transparent and conservative system in which local level stakeholders such as communities are rewarded for the (measurable) forest enhancement impacts of their management, rather than the (essentially non-measurable) avoided degradation. In the future, when sufficient data is available to construct credible reference emission levels for degradation, a decision would have to be made about to whom these avoided degradation credits pertain.

## 2. Discussion

When the concept of Reduced Emissions from Deforestation (RED) was first introduced to the UNFCCC (it was presented by the Brazilian research institute IPAM and Environmental Defense, at a side event at CoP11 in Montreal), the idea was simply to assess the rate of forest area loss and reward countries that were able to lower this. Very quickly however, a second D was adopted to include Degradation (REDD) for two main reasons. Firstly, it was evident that losses of carbon from within forests which remain forests may be high in many places. Secondly, many observers expressed the view that if degradation were not measured and included, leakage from avoided deforestation could occur. A related issue concerns the replacement of natural forest by tree plantations. The rapidly expanding oil palm plantations in Indonesia for example have a canopy cover of more than 30% and thus under UNFCCC terms may qualify as 'forests' [9]. Conversion of primary forest to oil palms, however, involves a loss of around 100-150 tons C per ha [10,11]. Under REDD+ accounting, any future land use changes of this kind need to be defined as 'degradation' and the carbon losses included in national REDD+ accounts.

There has been some debate in the literature about how to define degradation [11,3,12]. The Marrakech Accords define forest as land use with tree cover of over 10-30%, with a height at maturity of 2-5 meters, and a minimum area of 0.1-0.5 hectares; countries select their own thresholds within these ranges. Deforestation is then implied when canopy cover falls below the selected threshold. Degradation is implied when there are losses of biomass, but the threshold is not reached. Sasaki and Putz [3] have argued for raising the threshold definition of forest to 40% on the grounds that if degradation is not included under REDD+, loggers will be able to reduce the density of forests down to 30% or even 10% without 'deforesting'. Parties have however made it clear that degradation must be included in the REDD+ agreement for the reasons given above.

Ecologists and conservationists [3,13] have rightly argued that in general terms, degradation involves many other forest values than simply carbon stocks, but for the purposes of REDD+ accounting it does need to be assessed primarily in terms of loss of biomass (and hence carbon stock) from forests that remain forests. A special report to the UNFCCC [14] concluded that a space and a time element are required for the definition of degradation. This is because, if a forest is managed on (say) a 20 year rotational felling system, in any one year areas cleared in that year could be considered deforested, while some of the re-growth areas (say after 10 years) might be considered forested (i.e. with > 30% canopy) but degraded (because in reference to the original forests, carbon stocks are lower, even though these stocks may currently be *increasing*). Taken as a whole the management unit may be stable as regards carbon content, although the average level of stocking would be lower than in the original vegetation (i.e. on average it is *degraded *but not *degrading *further). Similar difficulties may occur in areas where there are stable swidden farming systems in place. Penman et al. [14] were not able to provide the space and time thresholds because of the enormous variation in management systems that exists.

Cadman [15] suggests that to avoid this difficulty, forests should be defined as degraded simply if they hold less carbon than the original, natural vegetation, but there are huge variations in natural ecosystem stock levels due to micro-site level biophysical conditions (soil depth, altitude, aspect etc). These make this definition difficult to operationalise for measurement purposes, although there is some potential in this regard for geographic analysis and modeling techniques [16].

Definitional problems also arise with regard to the fact that not all degradation is anthropogenic. According to the IPCC 4^th ^Assessment report [17], forest fires, pests and climatic events such as drought, wind, snow, ice and floods affect about 100 m hectares of forest globally each year, which is more than 10 times the area affected by deforestation. Accounting for biomass losses due to forest fires is particularly problematic, as in some cases it is unclear whether these are natural or manmade [8]. The methodological problem of 'factoring out' is currently being discussed at the level of UNFCCC in connection with broader policy on Land Use, Land Use Change and Forestry, and REDD+ may benefit from the outcomes of this debate.

The importance of defining degradation is entirely related to how to include it in REDD+ accounting. Measurement is difficult unless there is clarity about constitutes degradation in practice.

### 2.1 Towards a practical definition of degradation for REDD+ measurement purposes

The following points may help to build locally suitable definitions of degradation which will allow measurement and accounting of the related carbon emissions:

• At the level of the forest management unit, degradation needs to be seen as a dynamic element, incorporating forest enhancement ('negative' degradation) in a balance of stock change. Sustainable management of forests may be considered a strategy to promote reduced degradation and forest enhancement.

• Within any forest management unit, positive and negative changes in carbon stock need to be defined and measured in the context of the locally operating long-run management plan or practice, rather than only over the accounting period.

• Factoring out of non-anthropogenic factors behind stock changes, such as natural fires, may in rare cases be required, though in most cases fires may be considered anthropogenic.

• Non-carbon parameters of degradation (e.g. reduced biodiversity, reduced infiltration, etc) would be better included as safeguards or secondary conditions that have to be met before credits for reduced degradation are issued, in much the same way as community welfare and biodiversity are included in the Verified Carbon Standards for sale of emission credits in voluntary carbon markets

• Defining different types of degradation is fundamental to measuring it adequately. Large geographic areas tend to be affected either by commercial logging or by community over-exploitation, rarely by both, although both may also be subject to manmade fire. As we noted in an earlier article, [18], very different MRV methods are applicable to these different forms of degradation.

### 2.2 Tackling community uses of forest for early action on degradation

Of the three different types of degradation identified, degradation that is brought about by community over-exploitation of forest for subsistence purposes^c ^is probably the easiest to combat in many countries in the short term, for reasons that are summarized in Table 1. Measures to reduce community induced degradation could perhaps in some cases also help to reduce losses by manmade fires.

**Table 1:** Relative difficulty of tackling different forms of degradation

Degradation due to:	Most common in	Measures available to combat	Actors involved	Opportunity costs to actors of reducing degradation	Likelihood of leakage	Likely time horizon for implementation
**Industrial and commercial selective logging**	Humid tropical forest	Enforcement of existing codes; Introduction of stricter codes; Sector wide agreements on SFM practices with industry; Real time monitoring of logging and rapid response facility; Creation of indigenous peoples' reserves, with leakage safeguards	Commercial timber concerns, both legal and illegal; in some cases, corrupt or complicit officials	High	High	Long term; political opposition may be strong

**Community over-exploitation for subsistence and local market**	Dry (savanna) forest, high altitude temperate forests	CFM programmes, PES programmes	Communities, facilitating NGOs	Low; in many cases CFM increases the supply of subsistence products	Low, since productivity increases may make up for lost production	Short to medium: greatest barrier may establishment of tenure and rights, but is acceptable politically in most countries at least in low value forests

**Manmade fires**	All forests	Obligatory fire controls in SFM and CFM agreements; Real time monitoring and rapid response facilities	Communities, logging companies, other forest managers	Medium	Low	Long term; not least because the problem of factoring out natural fire from manmade is seriously difficult methodologically.

Many countries already have programmes aimed at improving community management of forests (CFM) - examples include Nepal, Tanzania, Vietnam, Mexico and India - and these have proven popular and relatively successful [19,20]. Such programmes typically involve a contract between the community and the state, giving the community rights over forest products provided these are extracted on a sustainable basis, under a very simple management plan or PES agreement^d^. The areas involved tend to be relatively small (50-500 hectares per community). In practice these programmes tend to result in reduced degradation rather than reduced deforestation, and importantly, forest restoration or enhancement (i.e. 'negative' degradation) is usually an additional outcome. The annual re-growth of biomass in forests recently brought under community management may in fact be as much as 3 - 5 times more than the annual degradation losses avoided [21]. In practice, three of the five REDD+ components (degradation, enhancement and SFM) are being addressed simultaneously in these kinds of programmes. As we have argued elsewhere [22], these components of REDD+ belong essentially to one cluster, both as regards management options and as regards MRV, while reduced deforestation and conservation may need a different set of management approaches and a different approach to MRV.

CFM is known to be relatively easy to implement. It is perceived as people-friendly, and compared to policies to control the main drivers of deforestation (Table 1) it requires only minor political shifts. Consequently, it has been put forward as an important element in many of the national level REDD Readiness plans submitted to the FCPF and to UN-REDD. In some countries, for example Tanzania, Mexico and Nepal, it is the central plank of the national strategy. However, the fact that community forestry management mainly addresses degradation, rather than deforestation, seems to have escaped the attention of the authors of most national REDD+ programmes. In selecting CFM as an option, degradation rates under improved management would have to be assessed against a baseline, not just at project level but also at national level, for inclusion in the national REL/RL and in national MRV systems. As we will try to show below, there are many unresolved challenges to be met in this regard.

### 2.3 The challenges of developing REL/RLs for degradation due to community uses of forest

There is a consensual view among most Parties to the UNFCCC and scientists working on REDD+ that national reference emissions levels should be based largely on historical data adjusted for national circumstances [2,23]. There are suggestions that such trends should be estimated for periods between 1990 and 2005. For assessing degradation and forest enhancement, baselines would be required both for the activity data and for the emission factors [18], since (in contrast with deforestation) degradation emission levels reflect the rates at which the standing stock has been diminishing in past years (which is highly site specific, and would need Tier 3 level data) and not the average per hectare stock in a standing forest (which could be derived at Tier 2 level, from secondary sources). If communities that adopt more sustainable management methods are to be incentivised via rewards for carbon savings, accurate estimates would in any case be needed at the level of the management unit. However, usually there are little or no historical data available at the local level on either the rate of spatial expansion of community-induced degradation or the per hectare annual losses, as there have been very few systematic forest inventories. Moreover carbon accounting for reduced degradation at the local level would have to produce data which could be 'nested' coherently, like a well fitting jigsaw puzzle, into the national reference level [24]. The data problems involved are summarized in Table 2.

**Table 2:** Data availability for RELs for community forestry and degradation

Level		Data available	Data not usually available
Community forest management projects registered for REDD+	Activity data	Area degraded at the start and at the end of the accounting period (derived from forest inventory and possibly modeling)	Historical rate of change of degraded area (not visible in medium resolution satellite images, high resolution images not available for earlier periods)
	Emissions factors	Stock change over the accounting period (monitored by forest inventory) Biophysical modeling Independently verified, qualitative assessment by experts that area had been degrading in the past.	Historical rate of biomass loss per hectare (not measurable from satellite imagery, no earlier forest inventories)

National level	Activity data	Would require geographic modeling over broad areas	Areas subjected to degradation by communities in the past, and rate of change of this area
	Emissions factors	Tier 2 level data on typical standing stock levels	Rate of change of standing stock

On the other hand the difficulty of obtaining activity data on this type of degradation at the national level is that the signature of degradation due to community uses is very difficult to obtain even from high resolution satellite imagery [18]. Moreover the typical mapping areas may be larger than the typical management units, so that scaling down would involve large errors due to averaging. Literature showing that rates of change of degraded area can be assessed using a combination of medium and high resolution images [e.g. [6]] relates only to disturbance due to selective logging and/or fire in rainforest. These impacts are more visible in satellite images because they tend to be concentrated in space and time. Over-exploitation by communities in contrast usually results in small losses per hectare spread thinly over very large areas, often manifested below canopy, which are therefore hardly discernible. At the national level, a possible solution to estimating the rate of expansion of degraded forest area would be to use geographical probability modeling, since degradation tends to correlate with population density, accessibility etc [25].

National historical emission factors for community induced degradation may be even more difficult to establish, since quantifying small per hectare/per year losses and gains of biomass from satellite imagery is even more difficult than identifying where such stock changes have been taking place. Modern technology such as Lidar could potentially do this in the future, but there are no Lidar images from the past that would be needed for reference. Moreover in most countries there is no database from forest inventory since these have not been systematically carried out in the past [18].

An alternative approach, which would avoid all these problems, would be to ignore past rates of degradation and simply to measure stock change within the management unit over the accounting period to establish a trend, by taking forest inventories at the start and end of the period. With historical reference set at zero, this would capture net increases in carbon stock monitored during the implementation period. It has been shown elsewhere that communities are able to make simple and reliable inventories [26,27]. Focusing only on above ground tree-based carbon pool (which is the largest and easiest pool to measure) will give conservative estimates of total carbon savings due to forest enhancement and reduced degradation (since earlier degradation must have been halted if there are increases in stock during the period measured) while simplifying data requirements. The fact that soil carbon stocks will also be protected (though not measured) is a further guarantee of a conservative estimate. Community based inventories would provide accurate, site specific Tier 3 level data for above ground biomass stock dynamics within such project sites, although the data would be patchy, and only available in areas which are under active management by communities (or other recognized organizations and individuals). The economic feasibility of this approach would of course depend on the relative cost of training communities and their carrying out of inventories, versus the value of carbon credits. The costs will however be lower than costs of inventories carried out by professional foresters [26], not so much because community labour is cheap, but rather because external expertise comes with a heavy transportation cost.

## 3. Conclusions

Community forest management offers a quick start option in national REDD+ programmes by which some types of degradation - particularly degradation that is caused by community over-exploitation of forests for livelihood purposes - can be tackled easily. The very widespread occurrence of this type of degradation means that carbon emissions, though low on a per hectare basis, are large in total. Implementing community forest management programmes to combat this type of degradation and to reverse it so that forest biomass is enhanced, does not require major policy changes in most countries and has low opportunity costs. It would however require reference emission levels.

Given the near impossibility of developing historical baselines for community induced degradation, the practical solution at the local level as regards rewarding community forest management and other similar initiatives would be to credit simply on the basis of positive stock changes during the accounting period, within any areas that are registered as being under management of this kind for REDD+. The only baseline that would be required would be a (qualitative) assessment conducted before the accounting period, to show that the forest had been degrading previously (otherwise the forest might have been increasing its stock under non-anthropogenically stimulated natural processes, continuation of which would not be additional). This approach to crediting would provide conservative estimates, since there would always be some (unmeasured, un-credited) avoided degradation present as well. The approach is transparent and credible, as it is based on real, measured increases in carbon sequestered, rather than counterfactual estimates of degradation that might have taken place in the absence of the project. Importantly, it would enable an early start to crediting, without the need to wait for complicated estimates of stock and area changes across the whole country in the past^e^. Seen from a national perspective, the approach would be unlikely to result in much leakage, except in cases where the earlier degradation was associated with supplies to consumers outside of the community, for example for the case of charcoal, as noted in endnote c. Such cases would clearly require special treatment.

At the national level, REDD+ involves all forest, not just areas actively engaged in CFM. It will take more time for management options for reduction of degradation due to selective logging and fire to be promulgated and to demonstrate their effectiveness in terms of reduction of carbon emissions. This will allow time for credible estimates of past degradation to be developed, for example using geographic modeling, so that in the future it may be possible to make claims for reductions in this degradation.

By limiting the crediting of community forest management efforts to forest enhancement, the problem of nesting of these credits with the national degradation reference level is resolved at least in the short term. Individual local level CFM projects benefit from enhancement credits immediately, while assessment of degradation is delayed until data allows credible reference emissions levels to be developed.

## Abbreviations

**CFM**: Community forest management; **FCPF**: Forest Carbon Partnership Facility of the World Bank, supporting country efforts in REDD+; **MRV**: Monitoring, reporting and verification as related to REDD+; **PES**: Payment for environmental services; **REDD+**: Reduced Emissions from Deforestation and forest Degradation; **REL**: Reference emission level; **RL**: Reference level; **UNFCCC**: United Nations Framework Convention on Climate Change; **UN-REDD**: A joint programme of UNDP, UNEP and FAO supporting country efforts in REDD+

## Competing interests

The authors declare that they have no competing interests.

## Authors' contributions

MS led the research, conceived the outline of the paper, and was responsible for the most of the text. ABT contributed written material on crediting and nesting, TM on the characteristics of locally-driven degradation, AG on remote sensing and modeling, and MH on the appropriateness of different MRV methods for locally-driven degradation. All five authors were involved in the final preparation of the draft and have read and approved the final manuscript.
